# Which patients are prone to suffer liver metastasis? A review of risk factors of metachronous liver metastasis of colorectal cancer

**DOI:** 10.1186/s40001-022-00759-z

**Published:** 2022-07-25

**Authors:** Mengdi Hao, Kun Wang, Yuhan Ding, Huimin Li, Yin Liu, Lei Ding

**Affiliations:** 1 Department of Oncology Surgery, Beijing Shijitan Hospital, Capital Medical University, Tieyilu 10 Yangfangdian, Haidian, Beijing, 100038 People’s Republic of China; 2 Department of Oncology Surgery, Ninth School of Clinical Medicine, Peking University, Beijing, China

**Keywords:** Colorectal cancer, Metachronous liver metastasis, Biomarkers, Risk factors

## Abstract

**Background:**

In recent years, with the increasing incidence of colorectal cancer (CRC) and its high fatality rate, CRC has seized the attention of the world. And liver metastasis, as the main cause of death of CRC, has become the leading cause of treatment failure in CRC, especially metachronous liver metastasis, have caused patients who underwent bowel resection to experience multiple tortures.

**Main body:**

Metachronous liver metastasis has severely affected the quality of life and prognosis of patients. Therefore, in this review, we discuss risk factors for metachronous liver metastasis of CRC, which is the premise for effective intervention for CRC patients who suffer metachronous liver metastasis after undergoing surgery, as well as the signaling pathways associated with CRC.

**Conclusion:**

The occurrence of metachronous liver metastasis is closely related to histology-based prognostic biomarkers, serum-based biomarkers, tumor microenvironment, pre-metastatic niche, liquid biopsy and tissue-based biomarkers. Further research is required to explore the risk factors associated with liver metastasis of CRC.

## Introduction

Due to globally increasing morbidity and mortality, more and more attention has been paid to colorectal cancer (CRC). According to GLOBOCAN 2020 statistics, CRC ranks the third most common malignancy in incidence, with more than 1.9 million new cases, whereas the second in cancer-related deaths [1], attributed to metastatic lesions. In general, the liver is the most site for CRC metastasis. Even during the disease course, more than half of CRC cases inevitably develop liver metastasis, of which synchronous liver metastasis may account for 25%. Among 20% of cases without initial metastasis, 1% would develop liver metastasis during one-year follow-up, whereas 15% during 5-year follow-up [2–7]. The median survival time (MST) of liver metastasis is generally no more than 12 months, even with aggressive treatment, MST would not exceed 13–18 months [3, 8–10]. With the application of targeted therapy using antibodies, MST of liver metastasis is expected to be gradually prolonged. Meanwhile, indicators such as tumor stage, genetic mutations, and lymph node involvement have been proposed to predict prognosis of CRC. However, liver metastasis remains challenging for CRC therapy. There is no consensus as to risk factors for liver metastasis of CRC. For newly diagnosed CRC patients without metastasis, clarifying potential risk factors for liver metastasis is paramount as it could have important clinical implications. In this review, we discuss the risk factors for metachronous liver metastasis in colorectal cancer. The reader is advised to refer to tables for the biomarkers and representative studies discussed in this review (Tables 1 and 2).

**Table 1:** Overview of the studies that have addressed risk factors of the metachronous liver metastasis of CRC

Tool citations	Sample size	Population/sample	Dates of data collection	Duration of follow-up	Final variables in model
Cheng et al. [126]	1969	CRC treated with surgery	2000–2013	8–163.4 months (median 36.3 months)	Patients with the BRAFV600E mutation are prone to non-regional lymph node metastasis and peritoneal metastasis, not liver metastasis
Feng et al. [121]	281	Primary tumor resections (R0)	2002–2008	In metachronous metastasis group, the median follow-up time was 87 months	Sex, primary tumor location, primary N stage and KRAS codon 13 mutations were independent factors for metachronous distant metastasis
Cho et al. [117]	147	Confirmed CRC by pathology and imaging studies confirmed metastatic disease	2007–2014	Unknown	KRAS and BRAF mutation have no correlation with liver metastasis of colorectal cancer and non-CEA producers are associated with RAS mutations
Margonis et al. [123]	849	Patients underwent resections with curative intent	2000–2016	28.3 months (median follow-up)	Mutbraf/wtkras genotype were also significantly more likely to be right-sided, more advanced T stage and metachronous liver metastasis
Colloca et al. [48]	425	Patients who diagnosed with relapsed or metastatic CRC	2006–2011	Unknown	1. Patients with synchronous metastasis: older, more frequent liver involvement, more right-sided primary tumors. 2. High CEA levels were related with synchronous liver metastasis
Tsai et al. [19]	155	Only CRC patients whose metastasis were resectable on presentation were included	1995–2004	Mean 28.5 ± 2.0 months	1. The metachronous group: the mean age was higher. 2. No significant difference between the synchronous and metachronous groups in terms of tumor location, tumor size, tumor staging, tumor grading and metastasis to regional lymph nodes
Mekenkamp et al. [14]	550	Only patients with a prior resection of the primary tumor were considered	2003–2005	Follow-up after completion of treatment was performed every 3 months until death. The primary endpoint was overall survival	Tumors of patients with synchronous metastasis had larger diameters, a higher T and N stage, absent or little lymphoid reaction and more frequently a diffuse infiltration pattern than patients with metachronous disease
Khan et al. [39]	434	Patients with histologically proven rectal carcinoma	2005–2015	5 years	The risk factors of metachronous group: tumor depth (T stage), lymph node metastasis, post-op serum CEA levels and complete tumor response on histopathology
Chuang et al. [20]	1099	Patients with histologically proven CRC receiving surgical treatment	2001–2007	Mean follow-up time of 39.0 ± 24.2 months	> 65 years, reoperative serum CEA level > 5/ml, tumor depth of T3–4 invasion, positive LN metastasis, positive vascular invasion, and positive perineural invasion are related to metachronous liver metastasis
Zheng et al. [22]	161	Colorectal adenocarcinoma determined by pathologic evidence	2008–2014	Unknown	1. Metachronous group: elder 2. Synchronous group: larger in size, poorly differentiated, more frequently local advanced and lymph node positive, result in more and larger metastatic lesions
Laubert et al. [52]	920/120	Patients who underwent surgery for colorectal cancer	1993–2008	5 years	Factors related to metachronous group: aneuploidy and elevated CEA
Amara et al. [76]	124 /35	CRC	1995–2011	The duration of follow-up was calculated from the date of surgery to death	SDF-1/CXCR4 may enhance the liver metastasis causing poor prognosis
Schøler et al. [34]	23	Liver metastasis patients treated with curative intent	2015–2016	3 years	CtDNA detected within 3 months post-surgery is associated with a very high relapse risk of liver metastasis
Huang et al. [110]	205	Histologically proven synchronous or metachronous mCRC who received surgical treatment	2002–2012	The median follow-up time for the 205 patients was 30.2 ± 20.9 months	1. Positive EGFR expression has prognostic value only for patients with metachronous liver metastasis 2. KRAS mutation did not have prognostic value in patients with metachronous or synchronous CRC
Pantal et al. [109]	18	Fresh tissue specimens from liver metastasis of 18 patients who had undergone liver surgery	/	/	1. EGFR was overexpressed in metachronous group 2. COX-2 gene was over expressed in synchronous group
Pan et al. [140]	20	Blood samples	/	/	1. HER2 is an independent predictive factor for synchronous liver metastasis 2. HER2 may also be a risk factor for metachronous liver metastasis
Styczen et al. [141]	208	Cancer samples and tissue samples	/	/	HER2 and HER3 expression status in primary tumors, is closely associated with metachronous liver metastasis

*CRC* colorectal cancer; *CEA* carcinoembryonic antigen; *COX-2* cyclooxygenase-2; *mir-200c* micrornas-200c; *ctDNA* circulating tumor DNA; *EGFR* epidermal growth factor receptor; *BRAF* B-type RAF kinase

**Table 2:** Overview of risk factors associated with liver metastasis of CRC

Factors associated with metachronous liver metastasis	References
Sex	Feng et al. [121]
Age	Tsai et al. [19]
	Chuang et al. [20]
	Zheng et al. [22]
Primary tumor location	Feng et al. [121]
T stage	Khan et al. [39]
N stage	Khan et al. [39]
	Chuang et al. [20]
	Yamauchi et al. [59]
	Feng et al. [121]
Positive vascular invasion	Chuang et al. [20]
Serum CEA levels	Khan et al. [39]
	Chuang et al. [20]
	Laubert et al. [52]
KRAS and/or BRAF genotype	Feng et al. [121]
	Margonis et al. [123]
	Carmen et al. [106]
	Huang et al. [110]
	Pantal et al. [109]
Chemokine (receptors) and CTC	Amara et al. [76]
	Schøler et al. [34]
Primary tumor growth pattern	Wu et al. [27]
Serum mir-200c	Yuji et al. [87]
COX-2	Yamauchi et al. [59]
Chromosome abnormality	Laubert et al. [52]
HER2	Styczen et al. [141]
HER3	Styczen et al. [141]

Factors associated with synchronous liver metastasis	References
Age	Colloca et al. [48]
Primary tumor location	Colloca et al. [48]
Primary tumor size	Mekenkamp et al. [14]
	Zheng et al. [22]
Tumor grading	Zheng et al. [22]
T stage	Mekenkamp et al. [14]
N stage	Mekenkamp et al. [14]
	Zheng et al. [22]
Serum CEA levels	Colloca et al. [48]
Primary tumor growth pattern	Mekenkamp et al. [14]
COX-2	Pantal et al. [109]
HER2	Pan et al. [140]

No correlation	References
Primary tumor location	Tsai et al. [19]
Tumor size	Tsai et al. [19]
Tumor staging	Tsai et al. [19]
Tumor grading	Tsai et al. [19]
	Cheng et al. [126]
	Huang et al. [110]
	Cho et al. [117]

*CRC* colorectal cancer; *CEA* carcinoembryonic antigen; *COX-2* cyclooxygenase-2; *mir-200c* micrornas-200c; *CTC* circulating tumor cell; *BRAF* B-type RAF kinase

## Histology-based prognostic biomarkers

A large number of studies have demonstrated that patients with liver metastasis at the initial diagnosis have a poorer prognosis than those with metachronous liver metastasis [11, 12]. Even among patients undergoing repeated hepatectomies [13], MST of metachronous metastasis remains superior to synchronous metastasis. In contrast, a recent retrospective study [14] observed no difference in overall survival (OS) between synchronous and metachronous liver metastasis. However, since only patients with primary tumor resection were included in this clinical trial, liver metastasis might have already occurred at the time of initial diagnosis. This indicates that the prognosis of synchronous vs. metachronous liver metastasis might be modulated by liver resection, which remains to be explored and verified.

Lymph node status has been recognized as a prognostic factor for CRC. It is generally believed that primary tumors metastasize to local lymph nodes first, and then metachronous distant metastasis occurs through lymphatic system [15]. Unexpectedly, distant metastasis can also occur in patients with negative lymph nodes [16, 17]. Thus, molecular mechanisms of distant metastasis vary greatly, especially for liver metastasis. When compared clinicopathological features, synchronous liver metastasis had a higher N grade than metachronous liver metastasis [14]. Even though no sign of liver metastasis was identified at the initial diagnosis, if the intestinal tumor had a higher TN grade, postoperative liver metastasis would probably occur [18]. Some patients may develop symptoms later related to increased size and specific location of the primary tumor, which can delay timely diagnosis, leading to local and/or distant metastasis at the time of initial diagnosis. There was no statistical difference in lymph node involvement between patients with synchronous and metachronous liver metastasis [19]. Interestingly, T staging was earlier in metachronous than in synchronous liver metastasis [14]. Moreover, lymph node involvement and vascular invasion have been identified as risk factors for recurrent liver metastasis in patients who had undergone curative resection [20, 21].

In addition, most previous studies have suggested that the difference between metachronous and synchronous liver metastasis relied on location, size and differentiation of the primary tumor [14, 22]. Nevertheless, some studies identified no difference in clinicopathology, except relationship of primary tumor size with CEA [19, 23].

In 2001, Vermeulen et al. [24] proposed liver metastasis as a heterogeneous tumor and classified CRC liver metastasis into 3 growth patterns, i.e., pushing, desmoplastic and replacement, based on histological differences. Subsequently, international consensus guidelines of liver metastasis applied this new classification on histopathological growth patterns (HGPs) which differentiate cancerous from normal liver cells [25]. Importantly, in subsequent retrospective studies, HGPs of liver metastasis were closely related to original features of primary tumors. Expanding CRC was more likely to develop into desmoplastic liver metastasis; replacement liver metastasis was more likely to be caused by infiltration CRC. Compared with desmoplastic growth pattern, the prognosis of replacement growth pattern was much worse [26]. In addition, HGPs might correlate with gene expression of primary tumors [27]. For example, HGPs, low tumor budding score (TBS), and Crohn’s disease-like response (CDR) in combination with primary CRC could predict growth patterns of liver metastasis, and PIK13CA expression was upregulated in primary CRC with desmoplastic liver metastasis. More specific molecular biological principles remain to be explored.

## Serum-based biomarkers

### Carcinoembryonic antigen (CEA)

CEA, a tumor-associated antigen expressed on the surface of cancer cells originated from endoderm, is a structural protein of cell membrane. In CRC, CEA-positive rate is no less than 90% [28]. CEA has been recognized as an independent prognostic factor for CRC, associated with recurrence [23, 29–33]. Several studies identified no relationship of CEA with tumor stage and liver metastasis, due to limitations in sample size and statistical method [23, 34].

Regardless of different sites of recurrence or metastasis, CEA was closely related to the liver [33, 35, 36]. At present, CEA detection has become a routine procedure both before and after surgery, as an indispensable indicator to predict prognosis of CRC patients. There are many speculations about CEA-mediated tumor liver metastasis, and whether CEA is released into the blood by the primary tumor or the metastatic lesion is unclear. Primrose et al. [37] and Wang et al. [38] proposed that preoperative CEA level was an independent prognostic factor for CRC, however, if liver metastasis was the only predictor for adverse prognosis was unclear. In addition, preoperative serum CEA level was statistically significant with occurrence of metachronous liver metastasis [20]. Conversely, a retrospective cohort study enrolled 434 patients suffered from rectal cancer, and only postoperative serum CEA level was considered to be a risk factor for postoperative metachronous liver metastasis [39]. However, many previous studies proposed that both the preoperative and postoperative CEA levels indicated the tendency of CRC patients to develop systemic distant metastasis. Thus, preoperative increase in CEA might affect the spread of postoperative tumor. Significant increase in CEA after operation might affect the recurrence and survival of CRC [40–42].

As far as we know, CEA is eliminated in the liver, so as long as the metabolic function of the liver is impaired, a high level of serum CEA may present even in benign diseases [43, 44]. For example, in a mouse model of alcoholic liver disease, alcohol-damaged liver provides microenvironment for CRC liver metastasis through CEA-mediated inflammatory pathways [45]. For patients with pathologically confirmed CRC, CEA is produced by the primary tumor and released into the bloodstream, which then induces the production of proangiogenic factors in the liver tissue, affects the biological behaviors of proangiogenic endothelial cells, and participates in signal transduction in endothelial cells. CEA-mediated signaling pathways are conducive to microvascular invasion and distant metastasis [46, 47].

A retrospective analysis including 425 patients who diagnosed with relapsed or metastatic CRC reported that elevated CEA serum level was related to synchronous (but not metachronous) metastasis of metastatic colorectal cancer (mCRC) with poor prognosis [48]. However, undeniable association between metachronous liver metastasis and increased CEA has been widely recognized. Inevitable false negatives and false positives will cause confusion to accurate diagnosis of metachronous liver metastasis [49].

In recent years, combination of CEA with hydroxylated collagen peptide in urine has improved the sensitivity of detecting liver metastasis [50, 51]. The combination of CEA with aneuploidy (leading to changes in nuclear DNA content by rearrangement) may become a predictor of metachronous liver metastasis [52]. These new biomarkers are expected to be applied in clinical practice.

### Tumor microenvironment and pre-metastatic niche

Inflammatory bowel disease (IBD), a chronic non-specific intestinal inflammatory disease, is a common precancerous lesion with 10–18% chance of developing CRC [53–55]. In the context of IBD, excessive inflammatory cells infiltrate the intestinal wall. Inflammatory pathways are overactivated and inflammatory factors trigger a series of immune reaction. With the development of disease, tissue homeostasis unbalance occurs. Inflammatory factors provide a suitable environment for tumor growth, which greatly increases the possibility of dysplasia and malignant transformation of intestinal epithelial cells [54, 56, 57]. The development of serrated epithelial polyp from normal intestinal epithelium in response to prolonged inflammation subsequently enhances abnormal proliferation of intestinal tract [58]. In addition, inflammatory factors are involved in distant metastasis. A retrospective study performed immunohistochemistry on surgically resected CRC specimens and identified cyclooxygenase-2 (COX2), expressed only in tumor but not normal epithelial tissue, as a risk factor for metachronous liver metastasis [59].

Moreover, circulating inflammatory markers are associated with aggressiveness of CRC and may serve as predictors of metachronous liver metastasis [57, 60–62]. During tumor invasion, metastatic cascade is characterized by local invasion to adjacent tissues and consequent spreading to secondary organs [57, 63, 64]. Tumor cells are supported by microenvironment in order to proliferate and metastasize. Tumor cells, in turn, affect microenvironment of target organs before reaching the metastatic site. Specific microenvironment created in advance for subsequent metastasis is called pre-metastatic niche [65]. The specific mechanism of pre-metastatic niche is not yet clear. Before metastatic niche is formed in the liver, tumor cells need to break through the liver’s self-protection system, and prioritize inflammatory microenvironment, making tumor cells more prone to spread and invade. Therefore, increased inflammatory cytokines indicate a higher risk of liver metastasis. The liver host microenvironment plays an important role in tumor invasion and progression [45]. Furthermore, miRNAs transported by tumor-delivered exosomes (miRNAs-TEXs) are involved in establishing metastatic niches in the liver. Importantly, TEXs can predict metachronous metastasis [66].

Chemokines are chemotactic cytokines specifically bound to G-protein-coupled receptors, which can promote migration and colonization of inflammatory cells (such as white blood cells) towards tumor sites [67]. In CRC, macrophages stimulate the primary tumor to produce CXCL1, a member of CXC chemokines, binding to CXCR2 together with CXCL2, CXCL5, and CXCL8. CXCL1 promotes formation of tumor micro-vessels, as well as pre-metastatic niche upon positive feedback of CXCL1–CXCR2 axis, resulting in liver metastasis [68, 69]. CXCR4 is the most widely expressed chemokine receptor, activated after specific binding to CXCL12 (also known as SDF-1). CXCL12–CXCR4 participates in a variety of cellular activities, including tumor proliferation, survival, vascularization and metastasis, which plays a promoting role in developing liver metastasis in CRC [70–75]. No expression of SDF-1 was detected in normal liver tissue, while SDF-1 was expressed in primary tumor and liver metastasis in CRC [76]. Furthermore, CXCL16 is a risk factor for metachronous liver metastasis. CXCL8, CCL2 and CCL15 also correlate with occurrence and prognosis of distant metastasis of CRC [77–80].

As for tumor microenvironment in the liver, the density of mononuclear inflammatory cells infiltrating in primary tumor can be in proportion to that in metastatic lesion [81]. Although clinically common inflammatory indicators (CRP, lymphocytes, and CRP/lymphocytes) had no significant association with postoperative liver metastasis, they carried significance for prognosis [82, 83]. CRP was higher in CRC than that in control, however, IL-6 and CRP levels were not associated with liver metastasis [84]. Conversely, Lee et al. [85] and Calon et al. [86] supported relationship of IL-6 or IL-11 with CRC liver metastasis. In addition, during the formation of pre-metastatic niche in the liver, serum microRNAs-200c (miR-200c), prostaglandin E2 (PGE2), macrophage-colony stimulating factor (M-CSF) were upregulated [87–90]. Theoretically, pre-metastatic niche has already been established before tumor cells arrive at a specific metastasis site. However, it is difficult to detect pre-metastatic niche by conventional imaging in clinical practice. Therefore, to identify biomarkers involved in pre-metastasis niche and to discover tumor invasion as early as possible are guaranteed for timely treatment.

### Liquid biopsy

In recent years, liquid biopsy has gradually become a new alternative strategy to traditional biopsy, through real-time dynamic analysis of tumor composition. To minimize tumor heterogeneity in terms of space and time, liquid biopsy may overcome limitations of traditional biopsy. The most common liquid biopsy relies on circulating tumor cell (CTC) and circulating tumor DNA (ctDNA) [91–93]. ctDNA is a DNA fragment released from tumor into blood, carrying information on tumor genome. ctDNA is used for gene mutation analysis and tumor burden assessment [91, 94, 95]. Whether ctDNA is related to metachronous tumor metastasis [96–98] is debated [94].

Dispersal of a small number of tumor cells differs from metastasis. Metastasis can occur when highly heterogeneous mutation is initiated and a large number of tumor cells are disseminated. The number of metastatic tumor cells is actually very small and clusters of tumor cells are more likely to metastasize than individual one [95, 99]. CTC is continuously released during tumor development and progression. Similar to ctDNA, CTC may predict prognosis of colorectal cancer liver metastasis (CRLM). At present, CTC is applied to evaluate therapeutic efficacy of metastatic CRC, and to predict postoperative recurrence and survival [100, 101]. CTC is associated with CEA, and considered to be an important marker of CRLM [102, 103]. However, due to unknown cause-and-effect relationship, whether CTC can predict metachronous liver metastasis remains unclear.

## Tissue-based biomarkers

**Fig. 1:**
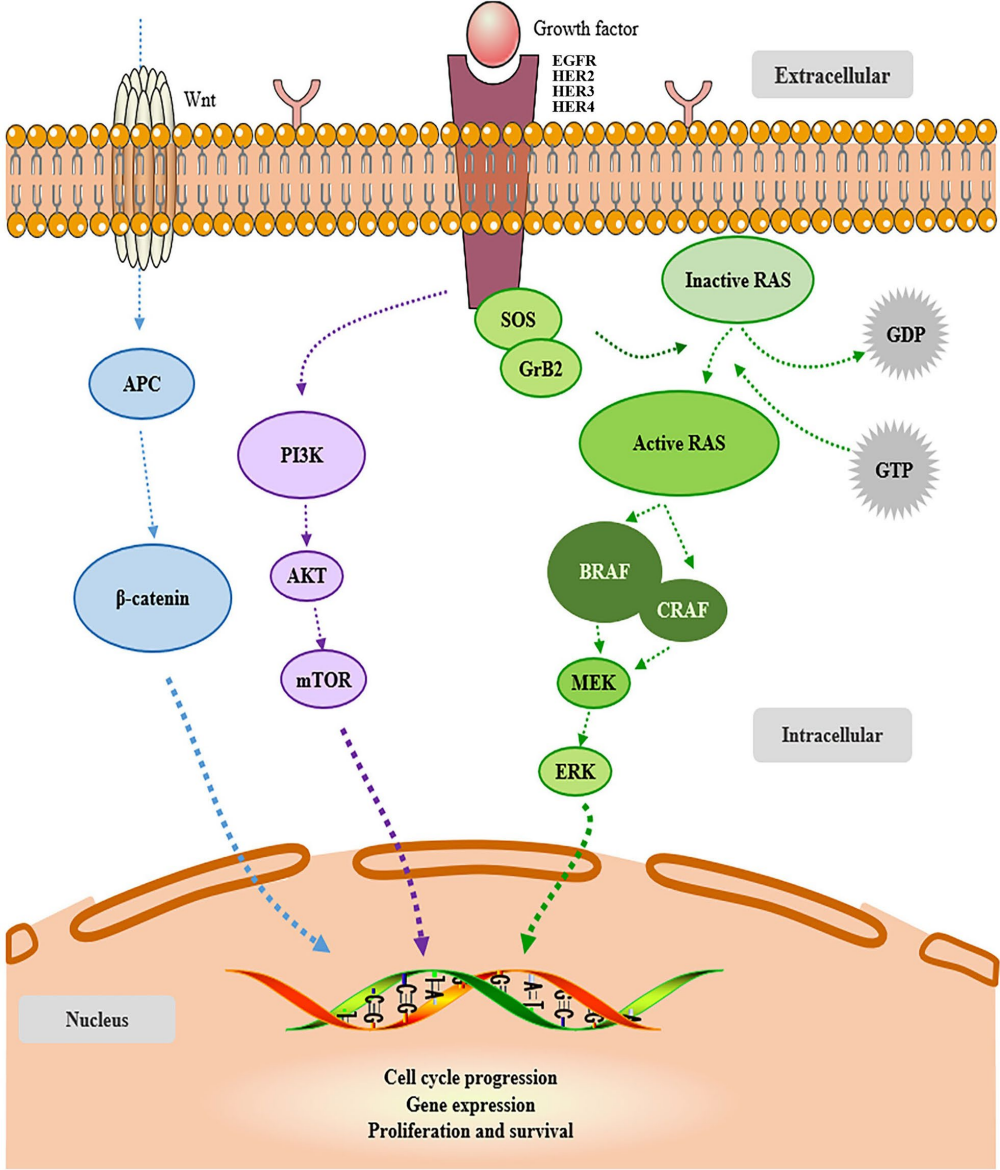
Overview of EGFR–RAS–RAF–MEK–MAPK pathway, a cellular signaling pathway involved in progression and proliferation of colorectal cancer

At present, selection of targeted therapy and evaluation of drug sensitivity vs. resistance can be achieved according to gene mutation landscape in combination with signaling pathways. Different gene mutational blueprints present in primary tumor and metastasis during tumor development and progression [104]. Furthermore, for synchronous and metachronous primary tumors, genotypes can vary substantially [105]. For CRC patients without initial metastasis at diagnosis or 6 months after surgery, genetic mutation signature may predict metachronous liver metastasis. Epidermal growth factor receptor (EGFR) is a receptor of epidermal growth factor (EGF), involved in cell proliferation and signal transduction. As a member of HER family, EGFR plays an important regulatory role in physiological processes. As a mitogen-activated protein kinase (MAPK) signaling pathway receptor, EGFR is overexpressed in CRC (Fig. 1). Upregulation of EGFR is observed in liver metastasis of CRC [106–108]. Furthermore, based on gene expression profile of liver metastasis, EGFR was overexpressed in metachronous liver metastasis of CRC [109], but not in synchronous liver metastasis [110].

### RAS

RAS gene family (Fig. 1), including KRAS, HRAS and NRAS, is an indicator of prognosis and therapeutic efficacy, with mutation rate of 35–45% in CRC [111, 112]. KRAS, in particular, is frequently mutated (25–52%) in CRC [5]. Mutations in RAS gene had no effect on CRC metastasis [113]. RAS gene expression was consistent between CRC and metastatic lesion. Probably, occurrence of metachronous liver metastasis may be predicted by postoperative genetic mutations [114]. Nevertheless, specific location and pattern of tumor metastasis related to KRAS are always disputed. For example, impact of KRAS mutation was detected in CRC distant metastasis, including liver metastasis [115]. KRAS mutation might more likely predict metastasis to the lung [116], rather than liver [117]. However, KRAS codon 13 mutation might play a role in CRC recurrence [118]. Furthermore, compared with KRAS codon 12, codon 13 mutation had poor prognosis, without distinguishing between metachronous and synchronous [119, 120]. KRAS codon 13 mutation is a risk factor for poor prognosis independent of metachronous distant metastasis [121]. KRAS codon 12 mutation was associated with synchronous metastasis.

### B-type RAF kinase (BRAF)

BRAF (Fig. 1), a component of MAPK signaling pathway, has a mutation rate of 8–12% in CRC metastasis, of which more than 90% was derived from V600E [4, 5, 111, 122]. BRAF is recognized clinically as a symbol of poor prognosis, with inferior survival rate. Only a few patients with BRAF mutations can undergo surgery. The MST of mutBRAF/wtKRAS genotype was 26 months [123]. In another clinical study, MST of BRAF-mutated CRC metastasis was only 10.4 months [124]. BRAF-related poor prognosis of CRC at different stages remains controversial. Price et al. [125], Tran et al. [124]. and Cheng et al. [126] reported that BRAF mutation affects prognosis of stage IV CRC. However, BRAF mutation was also associated with poor prognosis of stages II and III CRC [127]. Whether mutated BRAF can be a predictor of metachronous distant metastasis in CRC patients remains mysterious.

Margonis et al. [123] claimed that advanced T stage, metachronous liver metastasis and right-sided primary tumor were more likely caused by mutated BRAF/wild RAS. Non-V600E mutations might correlate with synchronous liver metastasis. Thus, BRAF mutations may be a risk factor for metachronous liver metastasis of CRC. Meanwhile, BRAF V600E mutation in CRC was more likely to occur on the right side [124, 126, 128, 129]. However, as far as we know, the right-sided primary tumor is more commonly to develop lymphatic spread and peritoneal metastasis, while the left-sided CRC is more prone to develop liver and lung metastasis. Similarly, Goldstein et al. [130] and Tran et al. [124] reported that BRAF mutation would increase the risk of lymph node and peritoneal metastasis. Due to relatively low mutation rate and insufficient sample size, patients with V600E mutations often have unresectable CRC tumors. Indeed, BRAF has limitations in predicting metachronous liver metastasis.

### Microsatellite instability (MSI)/microsatellite stable (MSS)

BRAF V600E mutation positively correlates with MSI, which is caused by loss of DNA mismatch repair (dMMR) expression [106, 111, 126, 131, 132]. Similar to BARF mutation, MSI is not very common in metastatic CRC. By contrast, dMMR was associated with favorable prognosis [111]. In pathologically diagnosed CRC, MSI had lower risk of liver metastasis compared with MSH [106] (caused by activation of Wnt/β-catenin signaling pathway) [133]. MSI is closely related to BRAF, so it is difficult to analyze the effect of MSI on liver metastasis of CRC separately from BRAF. Thus, cross-talk between BRAF and MSI requires further investigation.

### PIK3CA and TP53

PIK3CA (Pho-sphoinositide-3-kinase, catalytic, alpha polypeptide) is an important signal transduction factor downstream of EGFR (Fig. 1), with mutation rate of 20%-30% in CRC liver metastasis [27, 105, 134]. PIK3CA may complement and replace BRAF during tumorigenesis. Aggressiveness of CRC is positively associated with co-occurrence of PIK3CA over-activation and APC inhibition [111, 135]. TP53 is a tumor suppressor gene that regulates DNA damage repair and closely related to CRC development. At present, promoting role of TP53 alone in liver metastasis of CRC has not yet been confirmed, however, TP53 and RAS have a synergistic effect and jointly promote liver metastasis [111]. In all, the synergistic effects of signaling pathways in colorectal cancer jointly promote the occurrence of liver metastasis.

### HER2 and HER3

Among HER family members, besides EGFR, HER2, HER3 and HER4 also play an important regulatory role in the physiological functions of cells and the pathogenesis of solid tumors. HER2, in particular, is currently recognized as an oncogenic driver and has been proven to be one of the causative genes of breast cancer. The poor prognosis of breast cancer is associated with HER2/neu protein overexpression due to HER2/neu gene amplification, which is similar to colon cancer, and HER2 amplification is used in the treatment of CRC as one of the mechanisms of cetuximab resistance [136, 137]. Sawada et al. [138] analyzed the effect of HER2 status with BRAF and RAS status on the prognosis of mCRC and found that in terms of positivity rate, HER2 amplification was detected in a smaller proportion in RAS wild-type patients than in BRAF wild-type patients. In terms of OS, the ranking from highest to lowest was RAS mutation > HER2 amplification > RAS mutation and HER2 amplification synchronously > BRAF mutation. RAS/BRAF wild type has a better prognosis than HER2 amplification, and the latter has a greater correlation with the prognosis of metastatic CRC. In liver metastatic CRC, HER2 amplification is thought to be associated with younger age and left-sided RAS/RAF wild type [139]. In order to explore the molecules that may be involved in the mechanism of liver metastasis of CRC, Pan et al. [140] analyzed the serological levels of 24 molecules in peripheral veins and draining veins. Multivariate analysis showed that high peripheral blood HER2 level is an independent risk factor for synchronous liver metastasis, and may be a risk factor for metachronous liver metastasis. Although there is no uniform conclusion about the effect of HER2 expression on synchronous and metachronous liver metastasis, does this mean that HER2 expression in primary tumor state can be a key factor in predicting liver metastasis in CRC? It may provide us with new ideas.

In recent years, the expression of not only HER2, but also HER3 in CRC has gradually attracted attention. One study examined the expression status of HER3 in mCRC patients and concluded that there was a moderate correlation between HER3 expression in primary tumors and liver metastasis in CRC, and there was no difference in the expression of HER3 in synchronous and metachronous liver metastasis. Controversially, Styczen et al. [141] enrolled 208 patients with liver metastasis of CRC and analyzed the expression status of HER2 and HER3, suggesting that the expression status of HER2 and HER3 in primary tumors (especially HER3) is closely related to metachronous liver metastasis. HER2 lacks endogenous ligands, it relies on other EGFR family receptors to form heterodimers for activation, of which HER2/HER3 dimer is the most active and plays a core role in activating MAPK pathway and PI3K/AKT/mTOR pathway in cancer, HER3 overexpression is closely related to HER2, not only that, HER3 steadily plays a role in the progression of CRC [142–144].

At present, whether HER3 can be used as a predictor of metachronous liver metastasis is still in the initial stage of research. The high consistency of HER3 in primary tumors and liver metastasis provides a greater possibility for it to be a high-quality predictor.

## Conclusion and discussion

Clinically, the depth of primary tumor (T) invasion and lymph node (N) involvement have become indispensable indicators for predicting prognosis of CRC. In addition, tumor differentiation, site, venous or lymphatic invasion, as well as molecular biomarkers are associated with recurrence and prognosis of CRC [145–147]. The risk factors associated with metachronous liver metastasis, the most critical factors for postoperative prognosis of CRC, are a topic of ongoing attention by researchers. TN stage, lymph node involvement, vascular invasion, location, size, differentiation of the primary tumor, preoperative serum CEA level and postoperative genetics mutations are widely recognized as risk factors for metachronous liver metastasis, among which KRAS Codon 13 mutation and BRAF mutation are the most closely related indicators of metachronous liver metastasis. In addition to that, postoperative serum CEA level, the combination of CEA with aneuploidy, COX2, miRNAs-TEXs, CXCL1, SDF-1 and CXCL16 are also being considered as risk factors for metachronous liver metastasis. These biomarkers are still in clinical trials, but their similarity to CEA in the occurrence of liver metastasis is gradually being recognized. The application of circulating inflammatory markers and liquid biopsy to predict postoperative metachronous liver metastasis in CRC patients is imminent.

Although liver metastasis has been emphasized in CRC therapy, due to technical limitations, individual differences, and tumor heterogeneity, micro-metastasis is difficult to detect at the time of initial diagnosis. With the development of individualized treatment/precision medicine, adjuvant therapy may enable early diagnosis of liver metastasis. Currently, there is no consensus on defining synchronous or metachronous metastasis. Engstrand et al. [148] included a cohort of 1026 patients, respectively, defined 3-, 6- and 12-month post-diagnosis/surgery as cut-off points, and identified no significant difference in OS. Ueno et al. [149] defined metachronous liver metastasis as 12 months after primary surgery. Quireze et al. [12] and Mekenkamp el at. [14] proposed 6 months after the initial diagnosis of primary CRC as the time of diagnosis with metachronous liver metastasis. In this review, we advocate 6 months postoperatively as the time cutoff for metachronous liver metastasis.

Tumors are of polyclonal origin, which harbor spatial heterogeneity (uneven distribution of key molecular alterations across different regions) and temporal heterogeneity (variation in kind or arrangement of components across time) [150–152]. Genotypes should be determined from treatment [105]. For liver metastasis of CRC, diversity of primary tumor caused different biological behaviors, so primary and metastatic lesions are not identical [104, 105, 153]. Gene expression and molecular patterns of synchronous metastasis and metachronous metastasis are different. Synchronous liver metastasis is similar to local invasion, and is more inclined to become a disseminated disease [48]. Metastasis is a different disease state of advanced CRC, which is not the same as simple dissemination of molecules [19]. Moreover, some drugs are only effective for stage IV CRC, demonstrating heterogeneity of tumor biology at different stages [154].

At present, MAPK pathway is the major target for CRC liver metastasis treatment. Patients with metachronous liver metastasis usually receive chemotherapy or targeted therapy. Therefore, for patients who suffer from metachronous liver metastasis as demonstrated by imaging or symptoms, genetic mutation landscape, derived from various primary tumors or driven by secondary targeted therapy-induced mutations, is impossible to verify. Especially after receiving systemic chemotherapy for patients with no distant metastasis after operation, occurrence of metachronous metastasis and timing of metachronous metastasis will influence prognosis. In addition, targeted therapy may modify primary tumor tissue and serological indicators, and cause artificial interference to liver metastasis, and such error is unavoidable.

In conclusion, biomarkers and gene expression associated with metachronous distant metastasis of CRC can be reflected by primary tumor. To monitor patients after primary tumor resection can help early detect distant metastasis, especially liver metastasis. These biomarkers predict metachronous liver metastasis, so that patients' survival rate and quality of life may be improved. Furthermore, more mechanistic research is required to explore the progression of CRC and what factors accelerate the occurrence of metachronous distant metastasis.

## Data Availability

Not applicable.

## Abbreviations

CRC: Colorectal cancer
mCRC: Metastatic colorectal cancer
MST: Median survival time
OS: Overall survival
HGPs: Histopathological growth patterns
TBS: Tumor budding score
CDR: Crohn’s disease-like response
CEA: Carcinoembryonic antigen
IBD: Inflammatory bowel disease
COX2: Cyclooxygenase-2
miRNAs-TEXs: MiRNAs transported by tumor-delivered exosomes
miR-200c: MicroRNAs-200c
PGE2: Prostaglandin E2
M-CSF: Macrophage-colony stimulating factor
CTC: Circulating tumor cell
ctDNA: Circulating tumor DNA
CRLM: Colorectal cancer liver metastasis
EGFR: Epidermal growth factor receptor
EGF: Epidermal growth factor
MAPK: Mitogen-activated protein kinase
BRAF: B-type RAF kinase
MSI: Microsatellite instability
MSS: Microsatellite stable
dMMR: DNA mismatch repair
